# Current levels and needs of digital competence among medical teachers in Uzbekistan: a multi-institutional assessment and future directions

**DOI:** 10.1186/s12909-026-09288-3

**Published:** 2026-04-23

**Authors:** Hyojin Lee, Sanjarbek Khabibullaev, Sherzad Tuychiev

**Affiliations:** 1 BK21 Four Research & Education Center for Education, Korea University, Seoul, Republic of Korea; 2 Global Medical Education (g-MED) working group, Yonsei University Health System, Yonsei University, Seoul, Republic of Korea; 3 College of Medicine, Korea University, Seoul, Republic of Korea; 4 Tashkent State Medical University, Tashkent, Uzbekistan; 5 Department of Medical Humanities and Social Sciences, Medical School, Central Asian University, Tashkent, Uzbekistan

**Keywords:** Medical teacher, Medical education, Digital competence, Importance-Performance Analysis, Needs analysis, Professional development

## Abstract

**Background:**

Digital competence is essential for effective teaching in medical education, supporting technology integration and learner engagement. In low-resource contexts systematic evaluation of educators’ digital competence remains limited. This study assessed the digital competence of medical teachers in Uzbekistan and identified professional development priorities using the European Framework for the Digital Competence of Educators (DigCompEdu) and Importance–Performance Analysis (IPA).

**Methods:**

A cross-sectional online survey was conducted across four public medical institutes in Uzbekistan. Of 291 submissions, 266 valid responses were analysed. The questionnaire included demographic information and 22 DigCompEdu indicators across six competence areas. Participants rated perceived importance and self-reported performance on a five-point Likert scale. Based on performance scores, respondents were classified into DigCompEdu proficiency levels. IPA was used to examine gaps between importance and performance and to categorise competencies into four quadrants.

**Results:**

Overall, 66.9% of respondents were classified as “leaders” or “pioneers,” indicating high self-rated digital competence. Common strengths across proficiency groups included reflective practice, selecting digital resources, teaching with technology, learner engagement, and information and media literacy. Lower-rated areas included continuous professional development, creating and adapting digital resources, collaborative and personalised learning, digital communication, and responsible digital use. IPA revealed that less proficient teachers prioritised professional development and resource management, while intermediate groups emphasised supporting students’ self-regulated learning and problem solving. Among highly proficient teachers, creating and modifying digital resources and supporting learners’ digital content creation remained comparatively weaker areas.

**Conclusions:**

Medical teachers in Uzbekistan reported generally strong digital competence; however, gaps persist in competencies required for progression toward transformative digital pedagogy. Targeted professional development should prioritise digital resource creation, learner content creation, adaptive teaching practices, responsible digital use, and sustained opportunities for continuous professional development to enhance teaching quality in technology-mediated clinical education.

**Supplementary Information:**

The online version contains supplementary material available at 10.1186/s12909-026-09288-3.

## Introduction

Digital technologies have become an integral infrastructure of medical education, supporting content delivery, interaction, assessment, simulation, and telehealth training. MacNeill et al., [15], Masters, [17] Accordingly, the central question has shifted from whether technology can be adopted to how it can be integrated in ways that meaningfully enhance learning and educational impact. AMEE guidance has consistently emphasised that educational value depends less on the tools themselves than on pedagogically informed design, alignment with outcomes and assessment, and the sociotechnical context in which technologies are implemented ([7, 15]; Masters & Ellaway, [18]; [17]; O’Doherty et al., [21]). Evidence syntheses likewise suggest that digital education can support learning, but effects vary widely across modalities and implementation conditions ([27]; World Health Organization, [30]).

As technology becomes routine rather than exceptional, medical teachers require competence that extends beyond operational or technical skills. Recent studies highlight that ‘digital teaching’ draws on the integration of digital capabilities with pedagogical strategies and sound educational judgement, including the ability to select and use emerging tools appropriately [26]. The European Digital Competence Framework for Educators (DigCompEdu) offers a comprehensive, pedagogy-oriented conceptualisation of educators’ digital competence across professional engagement, digital resources, teaching and learning, assessment, learner empowerment, and facilitation of learners’ digital competence, and it describes a staged proficiency continuum from A1 to C2 [25]. This framework is therefore well-suited for profiling educators’ strengths and development needs in ways that are instructionally meaningful.

International policy initiatives further underscore the importance of building educators’ digital competence to support a resilient, future-ready health workforce. The World Health Organization (WHO) has highlighted the potential of digital education while noting that effectiveness depends on how digital methods are designed and implemented (World Health Organization, [30]). The rapid diffusion of AI and generative AI tools has intensified attention to ethics, transparency, data protection, and human oversight in medical education and clinical training [20, 28, 31].

Within the healthcare sector, digital competence encompasses dimensions that extend beyond general pedagogical applications of technology. Medical teachers operate at the intersection of education, clinical practice, and professional regulation, where digital tools increasingly shape learning environments, assessment practices, and professional norms. Consequently, educators are required not only to integrate digital technologies pedagogically but also to model responsible, ethical, and context-sensitive digital practices aligned with patient safety, data protection, and professional standards. In this respect, medical teachers represent a professional group whose digital competence influences both the quality of education and the preparedness of the future healthcare workforce (World Health Organization, [30]).

Despite increased policy attention and rapid digitalisation, technology integration in medical education remains uneven, with persistent infrastructure, organisational, and competency-related barriers [10]. Recent evidence syntheses continue to highlight persistent barriers to online learning in medical education, including infrastructure constraints, limited institutional support, and variability in educators’ readiness and confidence [3]. In Uzbekistan, the “Digital Uzbekistan–2030” strategy formalises national digital transformation, while post-pandemic experience highlights the importance of digital learning environments and staff support ([1]; President of the Republic of Uzbekistan, [24]). However, empirical evidence describing the digital competence profile of medical teachers and the areas where support is most urgently needed remains limited.

To inform feasible and targeted faculty development, approaches are needed that not only describe competence profiles but also prioritise improvement areas. While self-assessment instruments provide valuable insight into educators’ perceived digital competence, perceived proficiency does not necessarily translate into consistent pedagogical implementation, and descriptive profiling alone may offer limited guidance for strategic decision-making. Importance–Performance Analysis (IPA) enables comparison of perceived importance and performance to identify priority areas for improvement in educational practice [16, 29]. By directing attention to such discrepancies, IPA can support more focused and resource-sensitive faculty development planning. Moreover, because DigCompEdu frames competence development as progressive, pooled analyses may obscure stage-specific needs. A proficiency-stratified approach supports differentiated faculty development and aligns with transfer-oriented educational design [6].

Beyond contributing to the international medical education literature, this study is grounded in the practical objective of informing educational planning and decision-making at institutional and national levels in Uzbekistan. By providing an empirical profile of medical teachers’ digital competence across multiple public universities, the findings offer evidence to support faculty development planning, internal quality assurance processes, and alignment with ongoing national digitalisation strategies. Through formal institutional collaboration, the participating universities are positioned to engage with the results in order to inform locally relevant improvement initiatives.

### Aim of the study

This cross-sectional survey aimed to assess the digital competence of medical teachers at public medical institutes in Uzbekistan and to identify priority areas for improvement. Using DigCompEdu as the conceptual framework [25] and Importance–Performance Analysis (IPA) as the analytical approach [16, 22], we examined gaps between the perceived importance of digital competences and self-reported performance across DigCompEdu domains and items. To support differentiated professional development planning, we conducted IPA within DigCompEdu proficiency groups to identify level-specific priorities [32].

The research questions were formulated as follows: **RQ1.** How are medical teachers distributed across DigCompEdu proficiency levels? **RQ2.** Within each proficiency group, how do medical teachers perceive the importance of digital competences and their self-reported performance across DigCompEdu domains and items? **RQ3.** Which competences emerge as priorities for improvement within each proficiency group based on the IPA?

## Methods

### Characteristics of participants

The participants of the study were medical teachers who teach undergraduate medical students at four public medical institutes in Uzbekistan. In total, approximately 2,200 faculty members are employed across these institutes: 600 at the Tashkent Pediatric Medical Institute, 950 at the Andijan State Medical Institute, 650 at the Samarkand State Medical Institute, and 1,050 at the Tashkent Medical Academy. Each institute consists of about 40 departments covering pre-clinical, clinical, and elective courses, which constituted the target population of this study. Of the 291 responses received, 25 were excluded based on predefined data quality criteria established prior to analysis. Specifically, a response was excluded if it was incomplete or demonstrated uniform response patterns across all items (e.g., identical ratings throughout). Table 1 presents the demographic characteristics of the participants.

**Table 1:** Demographic information of the participants

Category		*n*	%
Gender	Male	124	46.6
	Female	142	53.4
Nationality	Uzbekistan	250	94.0
	Others	16	6.0
Age (years)	20 ~ 29	20	7.5
	30 ~ 39	113	42.5
	40 ~ 49	49	18.4
	50 ~ 59	51	19.2
	60 or older	33	12.4
Teaching experience (years)	0 ~ 5	107	40.2
	6 ~ 10	38	14.3
	11 ~ 15	27	10.2
	16 ~ 20	17	6.4
	21 or more	77	28.9
Academic position	Assistant professor	138	51.9
	Senior assistant professor	45	16.9
	Associate professor	60	22.6
	Professor	23	8.6

### Research instrument

The questionnaire consisted of two sections: demographic information and digital competence. The demographic section included items on gender, nationality, age, teaching experience, and academic position. The second section comprised 22 items aligned with the six areas of the DigCompEdu Framework: Professional Engagement, Digital Resources, Teaching and Learning, Assessment, Empowering Learners, and Facilitating Learner’s Digital Competence [25]. Each digital competence item was measured using a five-point Likert scale from 0 (*strongly disagree*) to 4 (*strongly agree*), yielding a total possible score between 0 and 88. In addition to rating their self-reported performance (P), respondents evaluated the perceived importance (I) of each item to enable Importance-Performance Analysis (IPA). Based on the total Performance (T) score, participants were classified into one of six proficiency levels of digital competence [14], as presented in Table 2. The internal reliability of the instrument was confirmed with the Cronbach’s Alpha of 0.98, indicating excellent consistency.

**Table 2:** Cut-off scores for digital competence proficiency levels [14]

Score range	Proficiency level
< 20	A1 – *newcomer*
20–33	A2 – *explorer*
34–49	B1 – *integrator*
50–65	B2 – *expert*
66–80	C1 – *leader*
> 80	C2 – *pioneer*

The questionnaire was based on the DigCompEdu self-assessment instrument developed by Redecker and Punie [25]. For the purposes of this study, the original English instrument in the framework was translated into Uzbek and Russian by an independent professional translator. To ensure content validity, two researchers from the research team, both native speakers of Uzbek and Russian and fluent in English, reviewed the translated items and compared them with the original version. Through iterative discussion and revision to address discrepancies, the research team reached consensus on the final translated version. The full English version of the survey questionnaire is provided as Supplementary Material 1.

The first area, *Professional Engagement*, covers teachers’ ability to use digital technologies appropriately and effectively in collaboration with students and colleagues to enhance teaching practice, and includes four items. The second area, *Digital Resources*, pertains to the responsible use and management of digital resources for educational purposes, and comprises three items. The third area, *Teaching and Learning*, encompasses the design, planning, and implementing the use of digital technologies within student-centred educational practices, and is represented by four items. The fourth area, *Assessment*, relates to the application of digital tools and strategies to evaluate student performance, diagnose learning needs, and improve instructional processes, and includes three items. The fifth area, *Empowering Learners*, pertains to the role of digital technologies in enhancing students’ motivation and participation by enabling differentiated and personalised learning experiences, and comprises three items. The sixth area, *Facilitating Learner’s Digital Competence*, extends beyond educators’ own competence to supporting learners’ digital competence development, and is represented by five items. The full list of DigCompEdu areas, items, and corresponding questionnaire statements is presented in Table 3.

**Table 3:** The questionnaire of digital competence [25]

Areas	Items		Questionnaire
1 Professional Engagement	organisational communication	1	I systematically use different digital channels to enhance communication with students, parents, and colleagues.
	professional collaboration	2	I use digital technologies to work together with colleagues inside and outside my educational organisation.
	reflective practice	3	I actively develop my digital teaching skills.
	digital continuous professional development	4	I participate in online training opportunities.
2 Digital Resources	selecting	5	I use different internet sites and search strategies to find and select a range of different digital resources.
	creating and modifying	6	I create my own digital resources and modify existing ones to adapt them to my needs.
	managing, protecting, and sharing	7	I effectively protect sensitive content, e.g. exams, students’ grades, personal data.
3 Teaching and Learning	teaching	8	I carefully consider how, when and why to use digital technologies in class, to ensure that they are used with added value.
	guidance	9	I monitor my students’ activities and interactions in the collaborative online environments we use.
	collaborative learning	10	When my students work in groups or teams, they use digital technologies to acquire and document evidence.
	self-regulated learning	11	I use digital technologies to allow students to plan, document and monitor their learning themselves.
4 Assessment	assessment strategies	12	I use digital assessment formats to monitor student progress.
	analysing evidence	13	I analyse all data available to me to timely identify students who need additional support.
	feedback and planning	14	I use digital technologies to provide effective feedback.
5 Empowering Learners	accessibility and inclusion	15	When I create digital assignments for students, I consider and address potential digital problems.
	differentiation and personalisation	16	I use digital technologies to offer students personalised learning opportunities.
	actively engaging learners	17	I use digital technologies for students to actively participate in class.
6 Facilitating Learner’s Digital Competence	information and media literacy	18	I teach students how to assess the reliability of information and to identify misinformation and bias.
	communication	19	I set up assignments which require students to use digital tools to communicate and collaborate with each other or with an outside audience.
	content creation	20	I set up assignments which require students to create digital content.
	responsible use	21	I teach students how to behave safely and responsibly online.
	problem solving	22	I encourage students to use digital technologies creatively to solve concrete problems.

### Data collection

The survey was conducted between September and November 2024. The questionnaire was developed using Google Forms. An official cooperation letter was first issued by the lead medical institute to the other participating institutions, requesting collaboration in disseminating the survey. Upon receipt of the letter, the vice-rector of each institution authorised the internal distribution of the survey to faculty members. The survey link and QR code were then circulated internally to medical teachers through official communication channels, including Telegram chatrooms, where all medical teachers were invited to participate. Participation was voluntary, and no financial or material incentives were provided. The study therefore relied on voluntary self-selection within the target population. A total of 266 valid responses were used for analysis.

#### Ethical approval

for the study was obtained from the Ethics Committee of the Ministry of the Republic of Uzbekistan (Approval No. 218–1863). All participants were informed about the purpose of the study and provided informed consent prior to completing the survey. This study was reported in accordance with the STROBE guidelines for observational research.

### Data analysis

Data were analysed using SPSS version 27.0. First, descriptive statistics were used to summarise participants’ demographic characteristics. Second, proficiency levels of digital competence were calculated by summing Performance (T) scores. Third, IPA was applied, incorporating paired *t*-tests for gap analysis and visualisation using an IPA matrix in the form of a Cartesian diagram.

**Fig. 1:**
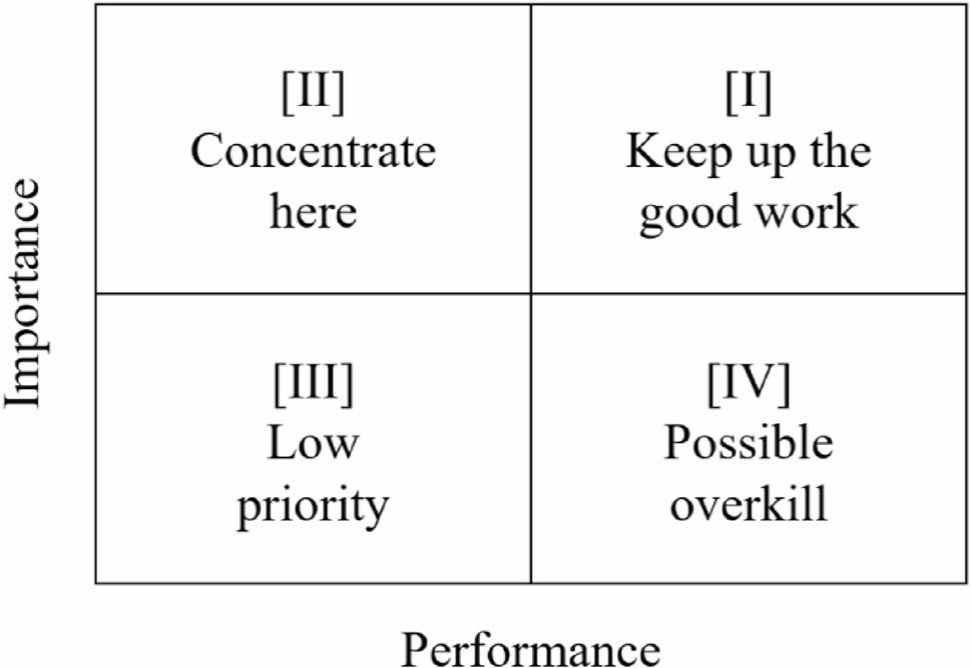
Importance – Performance Analysis (IPA) matrix [16]

The IPA matrix was divided into four quadrants by plotting the mean values of Performance (P) on the x-axis and the Importance (I) on the y-axis. Each item was analysed according to its position on the matrix to determine the level of priority for improvement. Items located in Quadrant I, ‘keep up the good work,’ were characterised by high importance and high performance, representing strengths that should be maintained. Quadrant II, ‘concentrate here,’ contained items of high importance but low performance, which were identified as the highest priority for improvement. Items positioned in Quadrant III were considered ‘low priority,’ having both low importance and low performance, indicating that minimal resources should be allocated to them. Finally, Quadrant IV, ‘possible overkill,’ included items with low importance but high performance, suggesting potential overinvestment of resources. The Importance–Performance Analysis matrix used in this study is illustrated in Fig. 1.

By examining the distribution of items within the IPA matrix, the analysis provides a practical tool for making efficient and evidence-based decisions about where to allocate resources, time, and effort for improvement. Statistical significance was determined at the *p* < .05 level.

## Results

### Distribution across DigCompEdu proficiency groups

As shown in Table 4, the 266 participants were categorised into three groups (A, B, and C) and six sub-groups according to the DigCompEdu proficiency levels. The overall mean score was 69.38 (*SD* = 19.12). Group A included 17 participants (6.4%), distributed across A1 *newcomer* (*n* = 2, 0.8%) and A2 *explorer* (*n* = 15, 5.6%), with an average of 23.94 (*SD* = 6.58). Group B comprised 71 participants (26.7%), divided into B1 *integrator* (*n* = 31, 11.7%) and B2 *expert* (*n* = 40, 15.0%), with a mean of 51.54 (*SD* = 10.05). The majority of participants (*n* = 178, 66.9%) were classified into Group C, distributed across C1 *leader* (*n* = 83, 31.2%) and C2 *pioneer* (*n* = 95, 35.7%), with a mean of 80.83 (*SD* = 6.77).

**Table 4:** Proficiency levels of medical teachers’ digital competence

Group	Proficiency levels	*n* (%)	M (SD)	Min.	Max.	Group	
						*n* (%)	M (SD)
A	A1 – newcomer	2 (0.8)	10.50 (7.78)	5	16	17 (6.4)	23.94 (6.58)
	A2 – explorer	15 (5.6)	25.73 (3.99)	20	31		
B	B1 – integrator	31 (11.7)	41.42 (4.01)	34	49	71 (26.7)	51.54 (10.05)
	B2 – expert	40 (15.0)	59.38 (4.94)	50	65		
C	C1 – leader	83 (31.2)	74.55 (4.22)	66	80	178 (66.9)	80.83 (6.77)
	C2 – pioneer	95 (35.7)	86.32 (2.35)	81	88		
Total		266 (100)	69.38 (19.12)	5	88	-	-

### Perceived importance and self-reported performance by each proficiency group

#### Group A

For Group A (*n* = 17), perceived importance ratings were consistently higher than self-reported performance across most items, indicating a general gap between expectations and implementation (Table 5). The most pronounced discrepancies were observed in facilitating learners’ use of digital technologies in collaborative learning and problem solving. In contrast, relatively smaller gaps were found in self-regulated learning and selected assessment-related items. Overall, the pattern suggests that less proficient teachers recognise the importance of digital competence but experience challenges in translating this awareness into practice.

**Table 5:** Descriptive statistics and paired *t*-tests for Group A (*n* = 17)

Areas	Items	Importance (I)	Performance (P)	Gap (I-P)	
		*M(SD)*	*M(SD)*	*M(SD)*	t*
1 Professional Engagement	1	1.88(1.05)	1.29(0.92)	0.59(0.94)	2.58*
	2	1.82(0.95)	1.24(0.83)	0.59(0.94)	2.58*
	3	2.00(1.17)	1.12(0.70)	0.88(1.22)	2.99*
	4	1.76(1.35)	0.94(0.66)	0.82(1.47)	2.31*
2 Digital Resources	5	2.12(1.17)	1.35(1.00)	0.79(1.15)	2.75*
	6	1.47(1.07)	0.71(0.59)	0.79(1.20)	2.63*
	7	1.94(1.35)	0.88(0.60)	1.06(1.52)	2.87*
3 Teaching and Learning	8	1.88(0.86)	1.35(0.49)	0.53(0.94)	2.31*
	9	1.65(1.06)	1.00(0.61)	0.65(1.17)	2.28*
	10	1.65(0.93)	0.88(0.60)	0.76(0.90)	3.49*
	11	1.41(0.87)	1.12(0.49)	0.30(0.77)	1.57
4 Assessment	12	1.65(1.12)	1.35(0.93)	0.30(0.59)	2.06
	13	1.71(0.99)	1.24(0.83)	0.47(0.80)	2.43*
	14	1.59(1.06)	1.06(0.69)	0.53(1.13)	1.94
5 Empowering Learners	15	1.59(1.12)	0.94(0.83)	0.65(1.12)	2.39*
	16	1.41(1.12)	0.76(0.44)	0.65(0.93)	2.86*
	17	1.76(1.03)	1.35(0.61)	0.41(0.94)	1.81*
6 Facilitating Learner’s Digital Competence	18	2.00(0.94)	1.24(0.97)	0.77(1.09)	2.89*
	19	1.35(0.70)	0.94(0.56)	0.41(0.80)	2.14*
	20	1.71(1.16)	1.12(0.86)	0.59(1.12)	2.16*
	21	1.53(1.23)	0.94(0.90)	0.59(1.18)	2.06
	22	1.94(1.14)	1.12(0.78)	0.82(1.07)	3.16*

*indicates statistical significance at *p* < .05 (paired *t*-test)

#### Group B

Group B (*n* = 71) demonstrated higher overall ratings than Group A, although importance scores remained consistently higher than performance across most competences (Table 6). Paired t-test results indicated statistically significant gaps for the majority of items. The largest discrepancies were observed in creating and modifying digital resources, considering when and why to use digital technologies in teaching, and supporting learners’ use of digital technologies for problem solving. In contrast, no statistically significant gaps were found in managing, protecting and sharing digital resources, facilitating collaborative learning using digital technologies, analysing evidence to support learning, and securing accessibility and inclusion.

**Table 6:** Descriptive statistics and paired *t*-tests for Group B (*n* = 71)

Areas	Items	Importance (I)	Performance (P)	Gap (I-P)	
		*M(SD)*	*M(SD)*	*M(SD)*	t*
1 Professional Engagement	1	2.49(0.95)	2.34(0.74)	0.16(0.55)	2.37*
	2	2.49(0.98)	2.31(0.80)	0.18(0.59)	2.60*
	3	2.63(0.93)	2.45(0.79)	0.18(0.52)	2.99*
	4	2.45(0.97)	2.20(0.75)	0.25(0.71)	3.00*
2 Digital Resources	5	2.65(0.96)	2.46(0.88)	0.18(0.49)	3.17*
	6	2.18(1.18)	1.86(0.93)	0.32(0.65)	4.20*
	7	2.86(0.96)	2.73(0.97)	0.13(0.58)	1.83
3 Teaching and Learning	8	2.89(0.84)	2.66(0.74)	0.23(0.51)	3.70*
	9	2.59(0.87)	2.41(0.80)	0.18(0.68)	2.26*
	10	2.46(0.97)	2.31(0.82)	0.16(0.71)	1.84
	11	2.54(0.92)	2.30(0.85)	0.24(0.57)	3.53*
4 Assessment	12	2.61(0.92)	2.44(0.84)	0.17(0.51)	2.81*
	13	2.65(0.86)	2.49(0.77)	0.16(0.67)	1.95
	14	2.44(1.04)	2.30(0.89)	0.14(0.52)	2.30*
5 Empowering Learners	15	2.62(0.82)	2.49(0.69)	0.13(0.58)	1.83
	16	2.30(0.93)	2.11(0.82)	0.18(0.54)	2.84*
	17	2.66(0.96)	2.46(0.92)	0.20(0.55)	3.02*
6 Facilitating Learner’s Digital Competence	18	2.72(0.97)	2.56(0.92)	0.16(0.50)	2.63*
	19	2.27(0.91)	2.11(0.84)	0.16(0.47)	2.79*
	20	2.18(1.13)	1.99(1.02)	0.20(0.73)	2.28*
	21	2.51(1.09)	2.25(1.00)	0.25(0.71)	3.00*
	22	2.58(1.01)	2.30(0.89)	0.28(0.66)	3.60*

*indicates statistical significance at *p* < .05 (paired *t*-test)

#### Group C

Group C (*n* = 178) exhibited the highest overall ratings for both importance and performance (Table 7). Although importance scores were generally higher than performance scores, paired *t*-test results indicated that the magnitude of these differences was relatively small. Statistically significant gaps were identified for most items; however, no significant differences were found in organisational communication, continuous professional development, selecting digital resources, managing, protecting and sharing digital resources, guidance in online environments, feedback and planning, and digital communication.

**Table 7:** Descriptive statistics and paired *t*-tests for Group C (*n* = 178)

Areas	Items	Importance (I)	Performance (P)	Gap (I-P)	
		*M(SD)*	*M(SD)*	*M(SD)*	t*
1 Professional Engagement	1	3.70(0.53)	3.66(0.57)	0.04(0.37)	1.40
	2	3.81(0.46)	3.74(0.51)	0.07(0.40)	2.45*
	3	3.81(0.46)	3.75(0.49)	0.06(0.30)	2.72*
	4	3.67(0.65)	3.62(0.70)	0.05(0.58)	1.17
2 Digital Resources	5	3.87(0.39)	3.84(0.41)	0.03(0.29)	1.29
	6	3.49(0.77)	3.31(0.87)	0.17(0.65)	3.56*
	7	3.80(0.49)	3.79(0.47)	0.01(0.25)	0.30
3 Teaching and Learning	8	3.85(0.39)	3.79(0.44)	0.06(0.32)	2.56*
	9	3.65(0.64)	3.64(0.63)	0.01(0.50)	0.30
	10	3.70(0.53)	3.61(0.57)	0.08(0.41)	2.74*
	11	3.79(0.45)	3.73(0.48)	0.06(0.31)	2.39*
4 Assessment	12	3.82(0.43)	3.79(0.49)	0.03(0.26)	1.74*
	13	3.75(0.52)	3.70(0.55)	0.05(0.32)	2.08*
	14	3.77(0.53)	3.74(0.54)	0.03(0.25)	1.51
5 Empowering Learners	15	3.70(0.53)	3.63(0.58)	0.07(0.45)	2.16*
	16	3.66(0.53)	3.57(0.62)	0.10(0.45)	2.85*
	17	3.82(0.44)	3.76(0.46)	0.06(0.30)	2.54*
6 Facilitating Learner’s Digital Competence	18	3.80(0.41)	3.76(0.47)	0.05(0.32)	1.90*
	19	3.64(0.61)	3.62(0.63)	0.02(0.42)	0.54
	20	3.57(0.61)	3.48(0.74)	0.10(0.54)	2.37*
	21	3.72(0.56)	3.61(0.68)	0.11(0.50)	3.02*
	22	3.76(0.48)	3.69(0.54)	0.07(0.41)	2.22*

*indicates statistical significance at *p* < .05 (paired *t*-test)

### IPA matrices and prioritised areas for improvement within each proficiency group

#### Group A

**Fig. 2:**
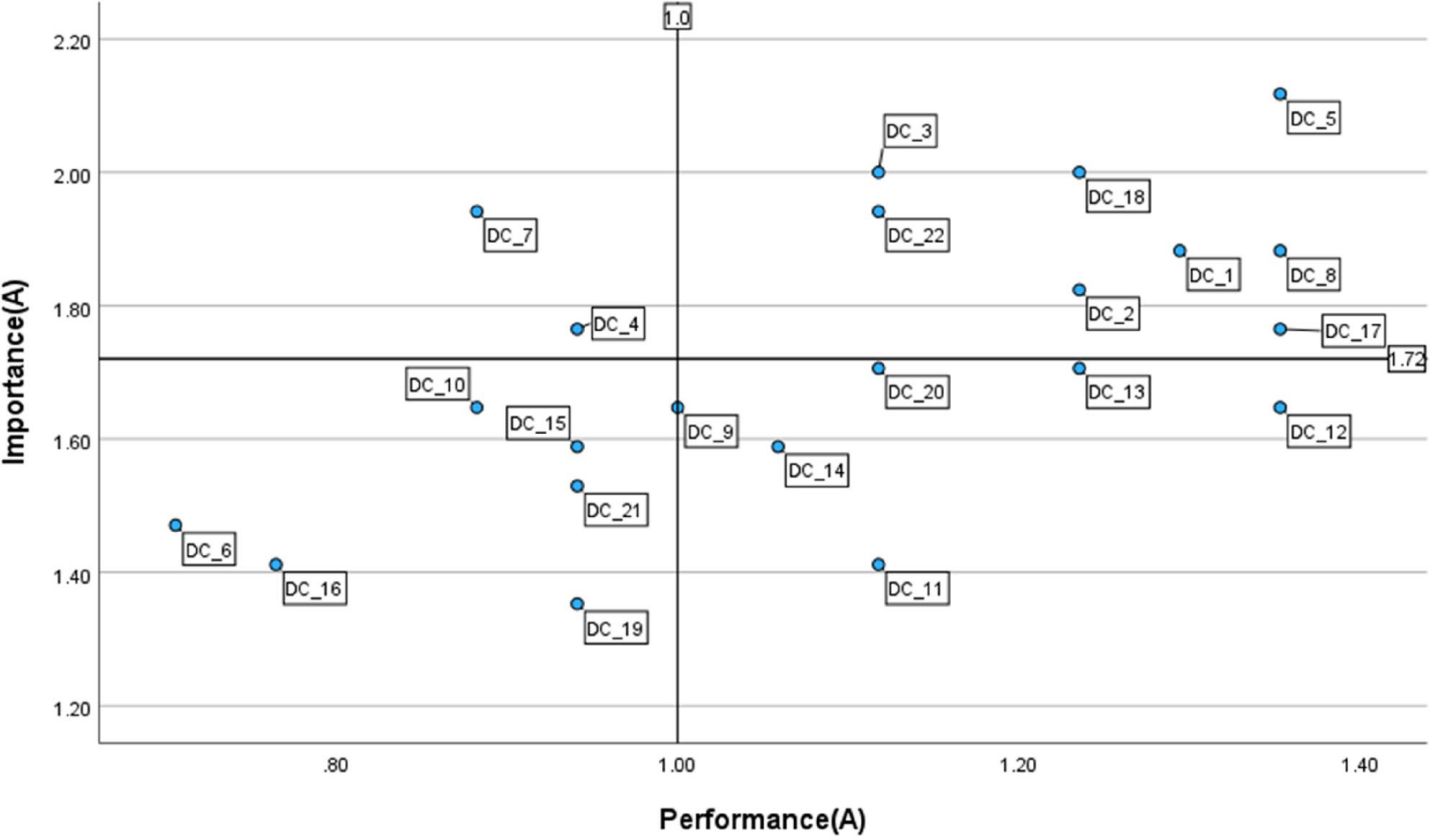
IPA matrix of DigCompEdu items for Group A (*n* = 17) Quadrant I = keep up the good work; Quadrant II = concentrate here; Quadrant III = low priority; Quadrant IV = possible overkill

As illustrated in Fig. 2, strengths area (Quadrant I) included items such as *organisational communication (1_1)*,* professional collaboration (1_2)*,* reflective practice (1_3)*, and *actively engaging learners (5_17)*. Priority areas for improvement (Quadrant II) were *continuous professional development (1_4)*, and *managing*,* protecting*,* and sharing (2_7)*. Several items, including *creating and modifying (2_6)* and *responsible use (6_21)*, fell into Quadrant III, while others such as *guidance (3_9)* and *feedback and planning (4_14)* were positioned in Quadrant IV, suggesting potential overinvestment.

#### Group B

**Fig. 3:**
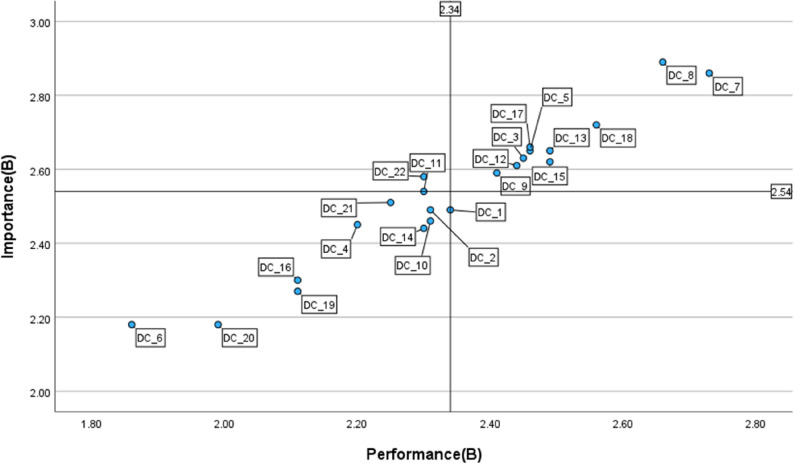
IPA matrix of DigCompEdu items for Group B (*n* = 71) Quadrant I = keep up the good work; Quadrant II = concentrate here; Quadrant III = low priority; Quadrant IV = possible overkill

Figure 3 illustrates the IPA distribution for group B, where strengths area (Quadrant I) included a wide range of items such as *reflective practice (1_3)*,* selecting (2_5)*,* teaching (3_8)*, and *actively engaging learners (5_17)*. In contrast, *self-regulated learning (3_11)* and *problem solving (6_22)* were allocated in Quadrant II, highlighting urgent needs for improvement. Nine items, including *professional collaboration (1_2)*,* continuous professional development (1_4)*,* creating and modifying (2_6)*,* feedback and planning (4_14)*, and *responsible use (6_21)*, appeared in Quadrant III. Only one item, *organisational communication (1_1)* was allocated in Quadrant IV.

#### Group C

**Fig. 4:**
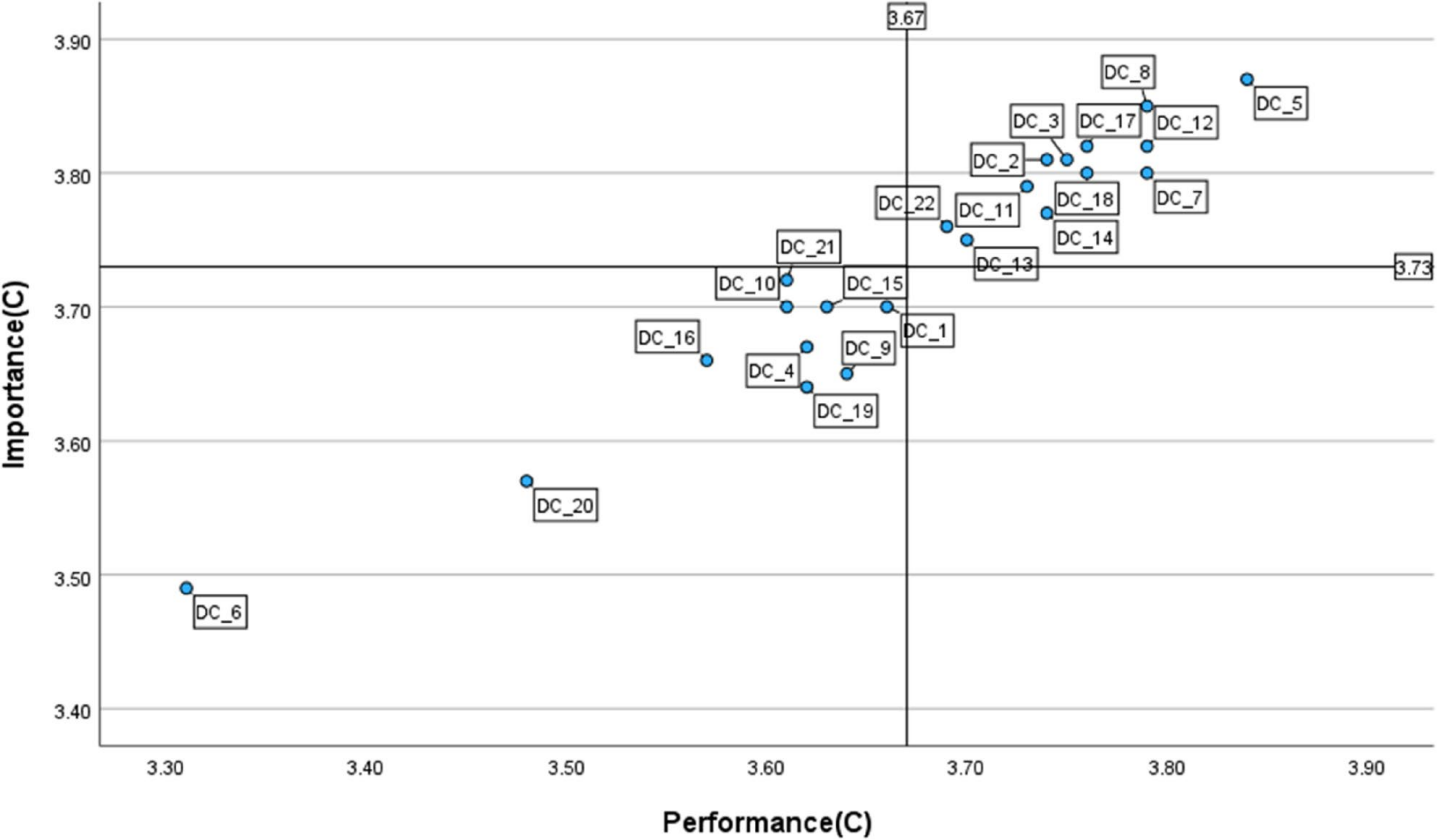
IPA matrix of the DigCompEdu items for Group C (*n* = 178) Quadrant I = keep up the good work; Quadrant II = concentrate here; Quadrant III = low priority; Quadrant IV = possible overkill

As illustrated in Fig. 4, Quadrant I contained twelve items, such as *reflective practice (1_3)*,* selecting (2_5)*,* teaching (3_8)*,* self-regulated learning (3_11)*, and *information and media literacy (6_18)*, indicating well-established strengths. Notably, no items were allocated in Quadrant II, suggesting no urgent needs for improvement. Quadrant III, however, included several items such as *organisational communication (1_1)*,* continuous professional development (1_4)*,* creating and modifying (2_6)*,* collaborative learning (3_10)*,* differentiation and personalisation (5_16)*,* content creation (6_20)*, and *responsible use (6_21)* that were perceived as lower in both importance and performance. No items appeared in Quadrant IV.

#### Comparative summary across groups

Table 8 provides a summary of IPA results across the three proficiency groups. Five items such as *reflective practice (1_3)*,* selecting (2_5)*,* teaching (3_8)*,* actively engaging learners (5_17)*, and *information and media literacy (6_18)* were consistently placed in Quadrant I across all groups, representing shared strengths. Conversely, six items including *continuous professional development (1_4)*,* creating and modifying (2_6)*,* collaborative learning (3_10)*,* differentiation and personalisation (5_16)*,* communication (6_19)*, and *responsible use (6_21)* consistently appeared in Quadrant III, reflecting lower priority areas of lower importance and performance. Group A identified *continuous professional development (1_4)* and *managing*,* protecting*,* and sharing (2_7)* as priority areas in Quadrant II, while Group B emphasised *self-regulating learning (3_11)* and *problem solving (6_22)*. In contrast, Group C had no items in Quadrant II, reflecting well-balanced perceptions of their own digital competences. These findings highlight both the common strengths of medical teachers across proficiency levels and the group-specific needs for targeted improvement strategies.

**Table 8:** Summary of IPA results for three proficiency groups

Category	Quadrant I (Keep Up Good Work)	Quadrant II (Concentrate Here)	Quadrant III (Low Priority)	Quadrant IV (Possible Overkill)
Group A	Organisational communication, Professional collaboration, Reflective practice, Selecting, Teaching, Actively engaging learners, Information and media literacy, Problem solving	Continuous professional development, Managing, protecting, and sharing	Creating and modifying, Collaborative learning, Accessibility and inclusion, Differentiation and personalisation, Communication, Responsible use	Guidance, Self-regulated learning, Assessment strategies, Analysing evidence, Feedback and planning, Content creation
Group B	Reflective practice, Selecting, Managing, protecting, and sharing, Teaching, Guidance, Assessment strategies, Analysing evidence, Accessibility and inclusion, Actively engaging learners, Information and media literacy	Self-regulated learning, Problem solving	Professional collaboration, Continuous professional development, Creating and modifying, Collaborative learning, Feedback and planning, Differentiation and personalisation, Communication, Content creation, Responsible use	Organisational communication
Group C	Professional collaboration, Reflective practice, Selecting, Managing, protecting, and sharing, Teaching, Self-regulated learning, Assessment strategies, Analysing evidence, Feedback and planning, Actively engaging learners, Information and media literacy, Problem solving	—	Organisational communication, Continuous professional development, Creating and modifying, Guidance, Collaborative learning, Accessibility and inclusion, Differentiation and personalisation, Communication, Content creation, Responsible use	—
Common items	Reflective practice, Selecting, Teaching, Actively engaging learners, Information and media literacy	—	Continuous professional development, Creating and modifying, Collaborative learning, Differentiation and personalisation, Communication, Responsible use	—

## Discussion

Overall, Uzbek medical teachers reported relatively strong digital competence, with shared strengths across proficiency groups in reflective practice, selecting digital resources, teaching with technology, actively engaging learners, and information and media literacy (Quadrant I). At the same time, several competences repeatedly appeared as lower-rated areas in the IPA matrices. In particular, continuous professional development, creating and modifying digital resources, collaborative learning, differentiation and personalisation, digital communication, and responsible use were frequently positioned in Quadrant III, indicating both lower perceived importance and lower self-reported performance. Although Quadrant III items are not considered urgent priorities within the IPA logic, their low perceived importance may signal under-recognised competences that warrant attention as medical schools move toward more transformative digital pedagogy. Similar findings have been reported in European and Nordic studies, where teachers often struggle to personalise digital learning, design original content, and address issues of online ethics and safety [4, 9, 23]. Continuous professional development was also rated relatively low: it emerged as an urgent priority for less proficient teachers (Quadrant II) and remained comparatively lower-rated among more proficient groups (Quadrant III). This is particularly concerning, as it implies that many teachers have few opportunities or little institutional encouragement to consistently develop their digital competences, a challenge also highlighted in low-resource contexts [11].

Group-specific analyses further contextualise these findings. Medical teachers in Group A (newcomers and explorers) identified continuous professional development and managing digital resources as urgent needs, reflecting their lower baseline competences and limited training experiences. This finding resonates with evidence that early-career or digitally inexperienced teachers require systematic institutional scaffolding to build confidence [12]. Group B (integrators and experts) emphasised self-regulated learning and problem solving as priority areas, indicating challenges in enabling students to take ownership of their learning and to apply technology efficiently and adequately to support such meaningful learning experiences. Research has shown that fostering students’ self-regulated learning through digital tools is one of the most complex areas of digital pedagogy, requiring sophisticated instructional design skills [8, 13]. Group C (leaders and pioneers), who comprised the majority, demonstrated a balanced competence profile with no urgent areas for improvement. Nevertheless, they still reported relatively lower performance in creating and modifying resources and in facilitating learners’ competence of creating digital content, echoing previous findings that even highly competent teachers often remain at functional rather than transformative uses of technology for themselves as well as for students [23, 25].

These findings have several implications for medical education in Uzbekistan. First, the recurring lower-rated areas suggest that professional development programmes should place greater emphasis on learners’ content creation, adaptive digital pedagogy, responsible use, and sustained opportunities for continuous professional development. Second, training should be differentiated by proficiency groups: foundational support for less competent teachers, pedagogical innovation for those at intermediate levels, and advanced, transformative practices for highly competent teachers. Such differentiation reflects international calls for personalised professional development pathways [4, 9]. Third, existing strengths such as reflective practice and collaborative engagement can be leveraged to foster peer learning communities that support the growth of weaker competences. Finally, strengthening teachers’ digital competence has broader implications for medical education, as digitally capable educators are better positioned to prepare students for practice in an increasingly technology-mediated healthcare environment, as outlined in frameworks of digital health competencies [5], systematic reviews of healthcare professionals’ digital readiness [2], and models of digital literacy development among health care academics [19].

In summary, this study provides evidence that Uzbek medical teachers perceived themselves as demonstrating relatively strong digital competence overall, with clear strengths in resource selection, reflective practice, and learner engagement. However, several competences were rated comparatively lower, particularly empowering learners; capacity of content creation, personalisation of learning, and responsible digital use. Addressing these challenges requires structured and differentiated professional development, institutional commitment, and the cultivation of a culture of continuous digital learning. By doing so, medical schools can enhance both the quality of teaching and learning and the digital preparedness of future healthcare professionals.

## Limitations and future research

Several limitations should be considered when interpreting these findings. First, this study relied on self-reported performance, which may not fully reflect educators’ actual use of digital technologies in classroom practice. Future studies should incorporate field-based, practice-oriented assessments (e.g., classroom observations, performance tasks, or portfolio-based evaluation) to capture digital competence in authentic teaching contexts. Second, because the survey captured teachers’ perspectives only, follow-up research should examine students’ perceptions of educators’ digital competence and how these perceptions relate to learning experiences; such evidence would strengthen the design of sustained, needs-based professional development. Third, the cross-sectional design limits inference about change over time, and self-selection into an online survey may introduce response bias. Longitudinal designs and mixed-method approaches could better clarify how competence develops and how contextual factors shape adoption. Finally, the sample was drawn from four public medical institutes in Uzbekistan, which may limit generalisability to other institutions or professional groups. Further studies should be conducted to replicate these findings in more diverse settings (e.g., private and regional institutions) and across other health professional groups.

## Conclusions

This study assessed the digital competence of Uzbek medical teachers using the DigCompEdu Framework and Importance–Performance Analysis (IPA). Although most teachers self-rated their proficiency as advanced, proficiency-stratified IPA revealed meaningful variability and distinct development priorities: newcomers and explorers prioritised continuous professional development and secure management of digital resources, whereas integrators and experts emphasised supporting students’ self-regulated learning and problem solving. Across groups, content creation, personalisation, and responsible digital use were comparatively lower-rated areas, suggesting the need for targeted, differentiated professional development to support progression from functional to more transformative practices. By applying the DigCompEdu Framework in a low-resource national context, this study underscores its practical value for mapping strengths and prioritising support while highlighting contextual challenges. Strengthening medical teachers’ digital competence is essential for improving teaching quality and preparing future healthcare professionals to thrive in increasingly technology-mediated clinical environments.

These findings carry several implications for medical educators, faculty developers, and institutional leaders. First, professional development programmes should be differentiated according to teachers’ proficiency levels rather than delivered uniformly, as the competence priorities identified for Groups A, B, and C diverge substantially. For less proficient teachers, foundational support in continuous professional development and secure management of digital resources may be most impactful, while more advanced groups would benefit from targeted capacity-building in supporting student self-regulated learning, problem solving, and digital content creation. Second, institutions should consider embedding digital competence development within existing structures, such as departmental workshops, peer mentoring, and community of practice, rather than treating it as a standalone training activity. Third, the comparatively low ratings for content creation, personalisation, and responsible digital use across all proficiency groups suggest that these areas warrant sustained attention in national-level faculty development strategies in Uzbekistan and similar low-resource contexts.

## Supplementary Information

Below is the link to the electronic supplementary material.

Supplementary Material 1.

## Data Availability

The datasets generated and/or analysed during the current study are available from the corresponding author on reasonable request.

## References

[1] Akhmedjanova D, Kerimova I. University Students and Teachers’ Experiences with Distance Education in Uzbekistan. J East Eur Cent Asian Res. 2024;11(1):156–75. 10.15549/jeecar.v11i1.1282.

[2] Alotaibi N, Wilson B, C., Traynor M. Enhancing digital readiness and capability in healthcare: a systematic review of interventions, barriers, and facilitators. BMC Health Serv Res. 2025;25. 10.1186/s12913-025-12663-3. Article 500. 10.1186/s12913-025-12663-3 PMC11969766 40186200

[3] Bastos RA, Santos Carvalho D, Brandão DR, Bergamasco CFS, Sandars EC, J., Cecilio-Fernandes D. Solutions, enablers and barriers to online learning in clinical medical education during the first year of the COVID-19 pandemic: A rapid review. Med Teach. 2022;44(2):187–95. 10.1080/0142159X.2021.1973979. 34608845 10.1080/0142159X.2021.1973979

[4] Caena F, Redecker C. Aligning teacher competence frameworks to 21st century challenges: The case for the European Digital Competence Framework for Educators (DigCompEdu). Eur J Educ. 2019;54(3):356–69. 10.1111/ejed.12345.

[5] Car J, Ong QC, Fox E, Leightley T, Kemp D, Švab SJ, Tsoi I, Sam KKF, Kent AH, Hertelendy FM, Longhurst AJ, Powell CA, Hamdy J, Nguyen H, Bahous HVQA, Wang S, Baumgartner M, Mahendradhata M, Popovic Y, Obadiel N, Y. A. The Digital Health Competencies in Medical Education Framework: An International Consensus Statement Based on a Delphi Study. JAMA Netw Open. 2025;8(1):e2453131–2453131. 10.1001/JAMANETWORKOPEN.2024.53131. 39888625 10.1001/jamanetworkopen.2024.53131

[6] Cecilio-Fernandes D, Sandars J, Gianotto-Oliveira R, Steenhof N. Teaching for transfer of learning in health professions education: AMEE Guide 176. Med Teach. 2025;47(8):1243–51. 10.1080/0142159X.2024.2414823. 39392459 10.1080/0142159X.2024.2414823

[7] Ellaway R, Masters K. AMEE Guide 32: e-Learning in medical education Part 1: Learning, teaching and assessment. Med Teach. 2008;30(5):455–73. 18576185 10.1080/01421590802108331

[8] From J. Pedagogical digital competence—Between values, knowledge and skills. High Educ Stud. 2017;7(2):43–50. 10.5539/hes.v7n2p43.

[9] Ghomi M, Redecker C. (2019). Digital competence of educators (DigCompEdu): Development and evaluation of a self-assessment instrument for teachers’ digital competence. In *Proceedings of the 11th International Conference on Computer Supported Education (CSEDU 2019)* (pp. 541–548). SCITEPRESS. 10.5220/0007679005410548

[10] Goh PS, Sandars J. A vision of the use of technology in medical education after the COVID-19 pandemic. MedEdPublish (2016). 2020;9:49. 10.15694/mep.2020.000049.1. 38058893 10.15694/mep.2020.000049.1 PMC10697445

[11] Gudmundsdottir GB, Hatlevik OE. Newly qualified teachers’ professional digital competence: Implications for teacher education. Eur J Teacher Educ. 2018;41(2):214–31. 10.1080/02619768.2017.1416085.

[12] Instefjord EJ, Munthe E. Educating digitally competent teachers: A study of integration of professional digital competence in teacher education. Teach Teacher Educ. 2017;67:37–45. 10.1016/j.tate.2017.05.016.

[13] Janssen J, Stoyanov S, Ferrari A, Punie Y, Pannekeet K, Sloep P. Experts’ views on digital competence: Commonalities and differences. Comput Educ. 2013;68:473–81. 10.1016/j.compedu.2013.06.008.

[14] Lucas M, Bem-Haja P, Siddiq F, Moreira A, Redecker C. The relation between teachers’ digital competence and personal and contextual factors: What matters most? Comput Educ. 2021;160:104052. 10.1016/j.compedu.2020.104052.

[15] MacNeill H, Masters K, Nemethy K, Correia R. Online learning in Health Professions Education. Part 1: Teaching and learning in online environments: AMEE Guide 161. Med Teach. 2024;46(1):4–17. 10.1080/0142159X.2023.2197135. 37094079 10.1080/0142159X.2023.2197135

[16] Martilla JA, James JC. Importance–performance analysis. J Mark. 1977;41(1):77–9. 10.1177/002224297704100112.

[17] Masters K, Correia R, Nemethy K, Benjamin J, Carver T, MacNeill H. Online learning in health professions education. Part 2: Tools and practical application: AMEE Guide 163. Med Teach. 2024;46(1):18–33. 10.1080/0142159X.2023.2259069. 37740948 10.1080/0142159X.2023.2259069

[18] Masters K, Ellaway R. e-Learning in medical education Guide 32 Part 2: Technology, management and design. Med Teach. 2008;30(5):474–89. 10.1080/01421590802108349. 18576186 10.1080/01421590802108349

[19] Matthews B. A model of digital literacy development in health care academics and the digital competency plexus. Discover Educ. 2025;4:218. 10.1007/s44217-025-00516-4.

[20] Ning Y, Teixayavong S, Shang Y, Savulescu J, Nagaraj V, Miao D, Mertens M, Ting DSW, Ong JCL, Liu M, Cao J, Dunn M, Vaughan R, Ong MEH, Sung JJ, Topol EJ, Liu N. Generative artificial intelligence and ethical considerations in health care: a scoping review and ethics checklist. Lancet Digit Health. 2024;6(11):e848–56. 10.1016/S2589-7500(24)00143-2. 39294061 10.1016/S2589-7500(24)00143-2 PMC11542614

[21] O’Doherty D, Dromey M, Lougheed J, Hannigan A, Last J, McGrath D. Barriers and solutions to online learning in medical education - an integrative review. BMC Med Educ. 2018;18(1):130. 10.1186/s12909-018-1240-0. 29880045 10.1186/s12909-018-1240-0 PMC5992716

[22] O’Neill MA, Palmer A. Importance-performance analysis: A useful tool for directing continuous quality improvement in higher education. Qual Assur Educ. 2004;12(1):39–52. 10.1108/09684880410517423.

[23] Pettersson F. On the issues of digital competence in educational contexts—A review of literature. Educ Inform Technol. 2018;23(3):1005–21. 10.1007/s10639-017-9649-3.

[24] President of the Republic of Uzbekistan. (2020, October 5). *On approval of the Strategy Digital Uzbekistan–2030 and measures for its effective implementation (Decree No. DP-6079).* LexUZ. https://lex.uz/en/docs/7008256

[25] Redecker C, Punie Y. (2017). *European framework for the digital competence of educators: DigCompEdu* (EUR 28775 EN). Publications Office of the European Union. 10.2760/159770

[26] Saaiq M, Khan RA, Yasmeen R. Digital teaching: Developing a structured digital teaching competency framework for medical teachers. Med Teach. 2024;46(10):1362–8. 10.1080/0142159X.2024.2308782. 38301620 10.1080/0142159X.2024.2308782

[27] Tudor Car L, Poon S, Kyaw BM, Cook DA, Ward V, Atun R, Majeed A, Johnston J, van der Kleij RMJJ, Molokhia M, Wangenheim FV, Lupton M, Chavannes N, Ajuebor O, Prober CG, Car J. Digital education for health professionals: An evidence map, conceptual framework, and research agenda. J Med Internet Res. 2022;24(3):e31977. 10.2196/31977. 35297767 10.2196/31977 PMC8972116

[28] UNESCO. Guidance for generative AI in education and research. UNESCO. 2023. 10.54675/EWZM9535.

[29] Wohlfart O, Hovemann G. Using importance-performance analysis to bridge the information gap between industry and higher education. Ind High Educ. 2019;33(4). 10.1177/0950422219838465.

[30] World Health Organization. (2021). *Global strategy on digital health 2020–2025.* World Health Organization. https://apps.who.int/iris/handle/10665/344249 10.2471/BLT.20.253955 PMC7133480 32284641

[31] World Health Organization. Ethics and governance of artificial intelligence for health: Guidance on large multi-modal models. World Health Organization; 2024. https://iris.who.int/handle/10665/376942.

[32] Yelon SL, Ford JK, Anderson WA. Twelve tips for increasing transfer of training from faculty development programs. Med Teach. 2014;36(11):945–50. 10.3109/0142159X.2014.929098. 24984563 10.3109/0142159X.2014.929098

