# Supplementary material for: Current levels and needs of digital competence among medical teachers in Uzbekistan: a multi-institutional assessment and future directions
Source: BMC Med Educ. 2026 Apr 23;26:922. doi: 10.1186/s12909-026-09288-3 (PMC13238038; doi:10.1186/s12909-026-09288-3)
Supplement: Supplementary file 1 — Supplementary Material 1. [file 12909_2026_9288_MOESM1_ESM.docx]

**DigCompEdu Questionnaire**

| 1 | I systematically use different digital channels to enhance communication with students, parents and colleagues. |  |
| --- | --- | --- |
| 2 | I use digital technologies to work together with colleagues inside and outside my educational organisation. |  |
| 3 | I actively develop my digital teaching skills. |  |
| 4 | I participate in online training opportunities. |  |
| 5 | I use different internet sites and search strategies to find and select a range of digital resources. |  |
| 6 | I create my own digital resources and modify existing ones to adapt them to my needs. |  |
| 7 | I effectively protect sensitive content, e.g. exams, students’ grades and personal data. |  |
| 8 | I carefully consider how, when and why to use digital technologies in class to ensure that they are used with added value. |  |
| 9 | I monitor my students’ activities and interactions in the collaborative online environments we use. |  |
| 10 | When my students work in groups or teams, they use digital technologies to acquire and document evidence. |  |
| 11 | I use digital technologies to allow students to plan, document and monitor their learning. |  |
| 12 | I use digital assessment formats to monitor student progress. |  |
| 13 | I analyse all data available to me to identify in a timely manner students who need additional support. |  |
| 14 | I use digital technologies to provide effective feedback. |  |
| 15 | When I create digital assignments for students, I consider and address potential digital problems. |  |
| 16 | I use digital technologies to offer students personalised learning opportunities. |  |
| 17 | I use digital technologies to enable students to actively participate in class. |  |
| 18 | I teach students how to assess the reliability of information and identify misinformation and bias. |  |
| 19 | I set up assignments that require students to use digital tools to communicate and collaborate with each other or with an external audience. |  |
| 20 | I set up assignments that require students to create digital content. |  |
| 21 | I teach students how to behave safely and responsibly online. |  |
| 22 | I encourage students to use digital technologies creatively to solve concrete problems. |  |

**DigCompEdu Questionnaire (Translated in Uzbek)**

| 1 | Oʻquvchilar, ota-onalar va hamkasblar bilan aloqalarni yaxshilash uchun turli raqamli kanallardan (ijtimoiy tarmoqlar, qo'g'iroq yoki SMS xabarlar) muntazam foydalanaman |
| --- | --- |
| 2 | Taʼlim muassasasi ichida va undan tashqarida hamkasblarim bilan birgalikda ishlash uchun raqamli texnologiyalardan foydalanaman |
| 3 | Raqamli texnologiyalardan foydalangan holda taʼlim berish koʻnikmalarimni faol rivojlantiraman |
| 4 | Onlayn treninglarda qatnashaman |
| 5 | Bir qator turli raqamli texnologiya resurslarini izlash va tanlash uchun turli internet saytlari va qidiruv platformalaridan foydalanaman |
| 6 | Oʻzimning shaxsiy raqamli texnologiyalarini yarataman yoki mavjudlarini oʻz ehtiyojlarimga moslashtirish uchun oʻzgartiraman |
| 7 | Imtihonlar, oʻquvchilarning baholari, shaxsiy maʼlumotlar kabi maxfiy ma`lumotlarni sir saqlayman |
| 8 | Samadorlikni taʼminlash maqsadida darsda raqamli texnologiyalardan qanday, qachon va nima uchun foydalanishni diqqat bilan ko‘rib chiqaman |
| 9 | Biz bog'lana oladigan onlayn platformalarda talabalarimning faoliyati va oʻzaro munosabatlarini kuzataman |
| 10 | Talabalarim guruh yoki jamoada ishlaganda, ma`lumotlarni toʻplash va rasmiylashtirish uchun ular raqamli texnologiyalardan foydalanishadi |
| 11 | Talabalar oʻzlarining taʼlim olishini rejalashtirishi, o'quv ma`lumotlarini olish va jarayonlarni kuzatishlariga imkon yaratadigan raqamli texnoligiyalardan foydalanaman |
| 12 | Talabalarning o'zlashtirish darajasini baholash uchun raqamli texnologiyalardan foydalanaman |
| 13 | Qoʻshimcha tushintirish va qoʻllab-quvvatlash kerak boʻlgan oʻquvchilarni oʻz vaqtida aniqlash uchun menda mavjud boʻlgan barcha maʼlumotlarni tahlil qilaman |
| 14 | Samarali fikr-mulohaza bildirish imkoniyati bo'lgan raqamli texnologiyalardan foydalanaman |
| 15 | Talabalarga online vazifa yaratyotganimda ehtimoliy muammolarni inobatga olaman va hal qilaman |
| 16 | Talabarlarga individual taʼlim olish imkoniyatlarini taklif qilish uchun raqamli texnologiyalaridan foydalanaman |
| 17 | Talabalarni darslarda faol ishtirok etishlari uchun raqamli texnologiyalardan foydalanaman |
| 18 | Talabalarga maʼlumotlarning ishonchliligini baholash, notoʻgʻri maʼlumotlar va noxolislikni aniqlashni oʻrgataman |
| 19 | Talabalarga bir-biri yoki boshqa insonlar bilan aloqa va hamkorlik qilishlarida qo'llanadigan raqamli texnologiyalarga oid topshiriqlarni beraman |
| 20 | Talabarlarga raqamli kontent yaratishini talab etuvchi vazifalarni beraman |
| 21 | Talabalarga internetda oʻzini qanday qilib xavfsiz va masʼuliyatli tutishlari kerakligini oʻrgataman |
| 22 | Talabarlani aniq muammolarni hal qilishda raqamli texnologiyalardan kreativ foydalanishga chaqiraman |

**DigCompEdu Questionnaire (Translated in Russian)**

| 1 | Я систематически использую различные цифровые каналы для улучшения качества общения со студентами, родителями и коллегами |
| --- | --- |
| 2 | Я использую цифровые технологии для совместной работы с коллегами как внутри, так и за пределами моей образовательной организации |
| 3 | Я активно развиваю свои навыки цифрового преподавания |
| 4 | Я участвую в расширении возможностей онлайн-обучения |
| 5 | Я использую различные веб-сайты и стратегии поиска, чтобы находить и выбирать различные цифровые ресурсы |
| 6 | Я создаю свои собственные цифровые ресурсы и модифицирую существующие, чтобы адаптировать их к своим потребностям |
| 7 | Я эффективно защищаю конфиденциальный материал, такой как материалы экзаменов, оценки студентов и личные данные |
| 8 | Я тщательно обдумываю, как, когда и зачем использовать цифровые технологии на занятиях, чтобы повысить эффективность учебного процесса |
| 9 | Я слежу за деятельностью и взаимодействием своих студентов в онлайн-среде, которую мы используем для совместной работы |
| 10 | Когда мои студенты работают в группах или командах, они используют цифровые технологии для сбора и документирования доказательств |
| 11 | Я использую цифровые технологии, чтобы позволить студентам самостоятельно планировать, документировать и контролировать свое обучение |
| 12 | Я использую цифровые форматы для оценки для отслеживания успеваемости студентов |
| 13 | Я анализирую все доступные мне данные, чтобы своевременно выявлять студентов, нуждающихся в дополнительной поддержке |
| 14 | Я использую цифровые технологии, чтобы обеспечить эффективную обратную связь |
| 15 | Когда я создаю цифровые задания для студентов, я выявляю и устраняю потенциальные цифровые проблемы |
| 16 | Я использую цифровые технологии, чтобы предлагать студентам персонализированные методы обучения |
| 17 | Я использую цифровые технологии, чтобы студенты активно участвовали в занятиях |
| 18 | Я учу студентов оценивать достоверность информации, чтобы выявлять ложные сведения и предвзятые суждения |
| 19 | Я предлагаю задания, которые требуют от студентов использования цифровых средств для общения и сотрудничества друг с другом или с внешней аудиторией |
| 20 | Я предлагаю задания, требующие от студентов создания цифровых материалов |
| 21 | Я учу студентов безопасной и ответственной работе в Интернете |
| 22 | Я призываю студентов творчески использовать цифровые технологии для решения конкретных задач |
